# Notes and News

**Published:** 1895-10-12

**Authors:** 


### Notes and News.

(Collected and Communicated.)

We are requested to state that the offices of the British Homes for Incurables, Streatham, have been removed to No. 72, Cheapside, E.C.

The Swanage Cottage Hospital, erected as a memorial to Mr. Ex-Sheriff Burt and his wife by their children, was opened on September 26th by the Bishop of Salisbury.

Her Majesty the Queen of Italy has presented to the Italian Hospital in London a portrait of herself bearing an inscription in her own handwriting expressing sympathy with the work carried on in the hospital, and appreciation of its value to Italians in London.

In conjunction with the Military Ambulance Service of France, a line is being constructed which will connect the Military Hospital at Vincennes with the railway. The ambulance carriages will be at the service of all the barracks in this part of France. Telephonic communication is being established between the barracks and the hospital, that the ambulance may attend where required.

Our prediction that the hospitals would reap a good harvest in 1895 appears to be verifying itself. The Lord Mayor has just received notice of a bequest from the late Mr. F. Waller of about £9,000, to be applied to the benefit of a hospital or hospitals for seamen situated near the river Thames. It appears probable that the Seamen's Hospital, Greenwich, as fulfilling the conditions, will reap the benefit. The addition to its funds will be most welcome, as the institution is much in need of funds. Under the same will the Gravesend Hospital receives £3,000. The Lord Mayor has also received towards the £4,000 needed to complete a collection of £50,000 for the Hospital Sunday Fund, £262 10s. from Messrs. Lewis and Marks, £100 from D. E. M., and £52 10s. from Mr. J. Pollak. The fund collection for 1895 closes on October 31st.

The new infirmary at Gosport was opened on September 26th by the Mayor of Southport, Alderman Dr. James Wood. It has been erected as a permanent memorial of the centenary of the town. It is intended to accommodate sixty patients. The building is of red brick, forming three sides of a quadrangle. There are special features in the construction. The cost has been £25,000, of which £4,000 still remains unsubscribed.

The Town Council of Cardiff have made an application to the Local Government Board to obtain leave to borrow £2,450 for the purpose of erecting a cholera hospital on the Flat Holm Island in the Bristol Channel. The existing hospital is a most unsuitable erection, capable of accommodating six patients only. This could be adapted as an adjunct to the new building, which, it is proposed, should consist of two wards each for six beds, lavatories, and nurse's room. The materials used in its construction would be stone and brick, with a slated roof. The Local Government Board are considering the application.

We understand that the Yarrow Home for Convalescent Children, Broadstairs, the formal opening of which lately took place, is now in perfect working order, and gradually filling up. The trustees are prepared to receive applications for admission of children, which should be addressed to the secretary of the home, at the London office, 73a, Queen Victoria Street, E.C. It should be understood that the institution is intended to benefit the children of middle-class or professional persons in adverse circumstances. The limits of age for the boys is from four to fourteen, for girls four to sixteen.

Dr. A. Brothers, of New York, has obtained the William F. Jenks Memorial Prize of 500 dollars for the best essay on "Infant Mortality during Labour, and its Prevention." The French Society of Public Medicine and Professional Hygiene offers three prizes for the best *mémoire* on "Preventable Diseases, and Preventive Measures to be taken." The essay must not exceed 10,000 words, and must include the consideration of the prevention of contagion during and after the attack; sanitation for private patients and for those who treat and tend them; house sanitation and disinfection, and general sanitation during illness. The first prize is to be 1,200 francs, the second 800 francs, and the third 500 francs.

The past year has been a busy one for the civil medical institutions of Bombay. According to the Surgeon-General's report the eleven hospitals of the city dealt with just under 111,000 cases, no less than 4,500 more than were treated during the same period in the previous year. Malarial fevers appear to be the most prevalent of these cases, and the Surgeon-General is not very hopeful of a diminution in fevers and other diseases until the drainage of the city is complete, the regulations as to overcrowding more efficiently enforced. Certain of these sanitary measures, we learn, are slowly but surely being carried out. At the Leper Asylum at Matoonga, 487 patients received treatment during the year. But as far as concerns the actual cure of leprosy, the numerous and highly recommended methods do not appear to bring us nearer attaining any really satisfactory results. The Salol treatment, it is stated authoritatively, has proved of use in checking leprous fever, but no instance of its efficiency as a cure has been instanced so far.

